# Hepatotoxicity and oxidative stress induced by *Naja haje* crude venom

**DOI:** 10.1186/1678-9199-20-42

**Published:** 2014-09-15

**Authors:** Saleh Al-Quraishy, Mahamed A Dkhil, Ahmed Esmat Abdel Moneim

**Affiliations:** Department of Zoology, College of Science, King Saud University, Riyadh, Saudi Arabia; Department of Zoology and Entomology, Faculty of Science, Helwan University, Cairo, Egypt; Department of Biochemistry and Molecular Biology, Asturias Institute of Biotechnology, University of Oviedo, 33006 Oviedo, Spain

**Keywords:** *Naja haje* venom, Hepatotoxicity, Oxidative stress, Apoptosis, Rats

## Abstract

**Background:**

Snake venoms are synthesized and stored in venom glands. Most venoms are complex mixtures of several proteins, peptides, enzymes, toxins and non-protein components. In the present study, we investigated the oxidative stress and apoptosis in rat liver cells provoked by *Naja haje* crude injection (LD_50_) after four hours.

**Methods:**

Wistar rats were randomly divided into two groups, the control group was intraperitoneally injected with saline solution while LD_50_-dose envenomed group was intraperitoneally injected with venom at a dose of 0.025 μg/kg of body weight. Animals were killed four hours after the injection. Lipid peroxidation, nitric oxide and glutathione levels were measured as oxidative markers in serum and liver homogenate. In addition, liver function parameters and activities of antioxidant enzymes were determined.

**Results:**

*N. haje* crude venom (0.025 μg/kg of body weight) enhanced lipid peroxidation and nitric oxide production in both serum and liver with concomitant reduction in glutathione, catalase, glutathione reductase and glutathione-S-transferase activities. Superoxide dismutase and glutathione peroxidase activities were significantly increased in liver of envenomed rats. These findings were associated with apoptosis induction in the liver. In addition, *N. haje* crude venom caused hepatic injury as indicated by histopathological changes in the liver tissue with an elevation in total bilirubin, serum alanine aminotransferase, aspartate aminotransferase, γ-glutamyl transpeptidase, and alkaline phosphatase.

**Conclusions:**

Based on the present results, it can hypothesized that *N. haje* crude venom is a potent inducer of toxin-mediated hepatotoxicity associated with apoptosis in the liver.

## Background

Snake venoms comprise complex mixtures that contain numerous different biological active compounds such as proteins, peptides and nucleotides. A number of these proteins interact with components of the human hemostatic system producing diverse effects [1].

The Elapidae family of venomous snakes – found in tropical and subtropical regions around the world – includes cobras, mambas, sea snakes and coral snakes [2]. Several species of cobras are natives to Africa, among them is the Egyptian cobra *Naja haje* (Linnaeus) found from southern Egypt to northern South Africa [3]. The venom of the Egyptian cobra consists mainly of neurotoxins and cytotoxins [4].

The venom of Egyptian cobra affects the nervous system by blocking the transmission of nerve signals to muscles and at later stages stopping those transmitted to the heart and lungs as well, causing death due to complete respiratory failure. Envenomation causes local pain, severe swelling, blistering, necrosis and variable non-specific effects [4]. Progress made over the past several decades has given rise to the identification of many of the venom components primarily responsible for these effects including phospholipases A_2_ and metalloproteinases. The former induces local myonecrosis and lymphatic vessel damage, whereas snake venom metalloproteinases (SVMPs) are responsible for local hemorrhage, extracellular matrix degradation, blistering and skin necrosis [5–7]. In addition, both PLA_2_s and SVMPs promote an inflammatory response that sets the stage for tissue repair and regeneration but, at the same time, may contribute to further tissue damage [8, 9]. Insights into the molecular structure of locally acting toxins has led to understanding their structure-function relationships and of the mechanisms involved in myonecrosis, hemorrhage, lymphatic vessel damage and dermonecrosis [5–7, 10–12].

Reactive oxygen species are involved in the inflammatory responses, thereby affecting the cellular physiology and playing a significant role in the pathological conditions [13]. The free radicals, apart from being involved in damaging cellular components, do play a significant role in venom induced toxicity [14].

Nevertheless, the effect of the venom of *N. haje* was not sufficiently covered in the available literature. Thus, it is of interest to examine the possible damaging effect of LD_50_ of the crude venom on liver of rats, unveiling the molecular mechanisms of venom-induced hepatotoxicity.

## Methods

### Experimental animals

Adult male Wistar albino rats weighing 180–200 g were obtained from The Holding Company for Biological Products and Vaccines (VACSERA, Egypt). Animals were kept in wire-bottom cages in a room under standard condition of illumination with a 12-hours light–dark cycle at 25 ± 1°C. They were provided with water and balanced diet *ad libitum*. We have followed the European Community Directive (86/609/EEC) and national rules on animal care that are in accordance with the NIH *Guide for the Care and Use of Laboratory Animals* (available at http://grants.nih.gov/grants/olaw/Guide-for-the-care-and-use-of-laboratory-animals.pdf).

### Venom source and chemicals

Ten specimens of *N. haje* were collected from the western Nile delta in Egypt, in September. They were kept alive in the laboratory at the University of Helwan in individual terrariums, fed fortnightly with mice and offered water *ad libitum*. Once a month, the snake venom was collected by milking. Then, it was diluted in deionized water, centrifuged at 10,000× *g* for 15 minutes and pellets were discarded. The sample was vacuum dried and stored at -20°C. Before use, the venom was reconstituted in saline solution, centrifuged at 3,000 rpm for ten minutes at 4°C and the supernatant was used in the present study. All solvents and chemicals used in this study were of analytical grade and deionized water was employed as well.

### Experimental protocol

LD_50_ of *N. haje* crude venom was determined as described by Meier and Theakston [15]. To study the effect LD_50_ of the crude venom on liver of rats after four hours, 12 adult male albino rats were randomly divided into two groups with six. The first group served as control and received intraperitoneally (IP) an injection of saline solution (0.2 mL saline/rat). The second group was injected IP with LD_50_ of *N. haje* venom in saline solution (25 μg/kg). Animals of the two groups were killed by cervical dislocation, and blood samples were collected by cardiac puncture. Blood was allowed to stand for half an hour and then was centrifuged at 500 *g* for 15 minutes at 4°C in order to separate serum and stored at -20°C until analysis. Pieces of the liver were weighed and homogenized immediately to give 50% (w/v) homogenate in ice-cold medium containing 50 mM Tris–HCl, pH, 7.4. The homogenate was centrifuged at 500 *g* for ten minutes at 4°C. The supernatant (10%) was used for the various biochemical determinations.

### Biochemical estimations

- **Liver function test**
  Colorimetric determination of alanine aminotransferase (ALT) or aspartate aminotransferase (AST) was estimated by measuring the amount of pyruvate or oxaloacetate produced by forming 2, 4-dinitrophenylhydrazine according to the method of Reitman and Frankel [16]. Moreover, serum γ-glutamyl transpeptidase (γGT) and alkaline phosphatase (ALP) were tested using kits purchased from Biodiagnostic Co. (Egypt) according to the method described by Szasz [17] and Belfield and Goldberg [18], respectively. Also, serum total bilirubin (TB) was assayed according to Schmidt and Eisenburg [19].
- **Determination of lipid peroxidation and nitric oxide**
  Lipid peroxidation (LPO) and nitrite/nitrate, as an indirect measure of nitric oxide (NO) production, were assayed colorimetrically in serum and liver homogenate according to the method of Ohkawa *et al.* [20] and Green *et al.* [21], respectively. LPO was determined by using 1 mL of trichloroacetic acid 10% and 1 mL of thiobarbituric acid 0.67% and were then heated in a boiling water bath for 30 minutes. Thiobarbituric acid reactive substances were determined by the absorbance at 535 nm and expressed as malondialdehyde (MDA) formed. Nitric oxide was determined in acid medium and in the presence of nitrite the formed nitrous acid diazotized sulfanilamide is coupled with N-(1–naphthyl) ethylenediamine. The resulting azo dye has a bright reddish-purple color that can be measured at 540 nm.
- **Estimation of glutathione and anti-oxidant enzymes**
  Glutathione (GSH) level was determined in serum and liver homogenate by the method of Ellman [22], which is based on the reduction of Ellman’s reagent [5,5′ dithiobis (2-nitrobenzoic acid) DTNB] with GSH in order to produce a yellow compound. The reduced chromogen was directly proportional to GSH concentration and its absorbance was measured at 405 nm. In addition, hepatic catalase (CAT) was determined colorimetrically according to the method of Aebi [23]. The assay is based on catalase-catalyzed reaction of a known quantity of H_2_O_2_ with 3,5-dichloro-2-hydroxybenzene sulfonic acid (DHBS) and 4-aminophenazone (AAP) to form a chromophore, which has a color intensity inversely proportional to the amount of catalase in the original sample which can be measured at 510 nm. Superoxide dismutase (SOD) activity was assayed by the method of Nishikimi *et al.* [24]. This assay relies on the ability of the enzyme to inhibit the phenazine methosulphate-mediated reduction of nitroblue tetrazolium dye. Also, activities of glutathione-S-transferase (GST), glutathione peroxidase (GPx) and glutathione reductase (GR) were determined by the methods of Habig *et al.* [25], Paglia and Valentine [26] and Factor *et al.* [27], respectively.

### RT-PCR analysis

Total RNA was extracted from frozen liver samples of six rats following the Trizol reagent method [28]. The extracted RNA was dissolved in water (diethylpyrocarbonate-treated) and stored at -70°C. Five μg of RNA was used as template for cDNA production through incubation with RevertAid™ H Minus Reverse Transcriptase Thermo Fisher Scientific Inc, Canada) for one hour at 45°C, in 10 lM random hexamers, 0.375 mM per dNTP, 3 mM MgCl_2_, 75 mM KCl, 50 mM Tris–HCl, pH 8.3, 10 mM dithiothreitol, and 40 units RNase inhibitor, followed by five minutes at 70°C to inactivate the enzyme. Samples were incubated for 30 minutes at 37°C with 0.1 mg/mL RNAse. PCR amplification was performed in the presence of 2 mM of MgCl_2_, 0.5 mM of each primer (Metabion International, Martinsried, Deutschland), 0.2 mM dNTPs, 2 U of Taq DNA polymerase (GoTaq™ DNA Polymerase, Promega Corporation) in a final volume of 25 μL. Simultaneous amplification of the invariant housekeeping gene GAPDH was performed. The sequences of the primers were as follows:

- iNOS (S): 5′-GAAAGAACTCGGGCATACCT-3′.
- iNOS (AS): 5′-GGCGAAGAACAATCCACAAC-3′.
- GAPDH (S): 5′-CAAGGTCATCCATGACAACTTTG-3′.
- GAPDH (AS): 5′-GTCCACCACCCTGTTGCTGTAG-3′

PCR conditions for iNOS consisted of 35 cycles of denaturation at 95°C for 45 s, annealing at 63°C for 45 s, and extension at 72°C for 45 s. PCR conditions for GAPDH were 25 cycles of denaturation at 95°C for 45 s, annealing at 60°C for 45 s, and extension at 72°C for 45 s. Following the last cycle, the final extension was performed at 72°C for ten minutes for all PCR analyses. PCR products were visualized on a 2% agarose gel with ethidium bromide staining. The expression of the tested enzyme was normalized to the expression of GAPDH of each sample and compared using TotalLab software.

### Flow cytometry

Liver tissue samples were prepared by manual disaggregation procedure. Briefly, a few drops of RPMI medium were added to tissue and then mixed until complete tissue disaggregation was achieved. Suspended cells were filtered using a 50-μm pore size mesh and then centrifuged at 1000 *g* for ten minutes. Cells were resuspended in PBS, counted and washed by calcium buffer then centrifuged at 1500 g for five minutes. The pellet was resuspended and then cells were counted. Annexin-PI apoptotic assay was carried out using BD Annexin V FITC Assay Kit (BD Biosciences, USA). FAC scan Becton-Dickinson (BD) flow-cytometer was used and data were analyzed using cell Quest software.

### Western blotting analysis

Western blotting analysis was performed according to the standard method. Briefly, cell lysates were prepared, separated on 12% sodium dodecyl sulfate polyacrylamide gels and transferred onto nitrocellulose membrane (Amersham Biosciences, USA). Non-specific reactivity was blocked by incubating the membranes for two hours in 5% bovine serum albumin at room temperature. Membranes were incubated with primary antibody (SOD, GPx, GR Bax, Bcl-2 or mitochondrial respiratory complexes namely, complex I, II, III and V) overnight at 4°C. After three washes for ten minutes with phosphate buffered saline tween-20 (PBST), the membranes were incubated at 37°C for one hour with the appropriate secondary antibody (1:5000 dilution) and washed three times with PBST. Reactive proteins were detected with the enhanced chemiluminescence (ECL) detection system (Pierce), β-actin was used as an internal control.

### Histopathological examination

Conventional techniques of paraffin-wax sectioning and hematoxylin-eosin staining were used for histological studies [29]. Pieces of fresh liver tissues were cut and fixed in neutral buffered formalin for 24 hours. Following fixation, livers were washed and processed through an ascending series of ethanol, cleared in methyl salicylate and infiltrated with wax at 57°C then embedded in paraffin. Sections of 5 μm were cut and stained with aqueous hematoxylin and alcoholic-eosin, then examined in a Olympus microscope at a magnification of 400 ×.

### Caspase-3 detection by immunochemistry

Immunolocalization technique for caspase-3 was performed on 3- to 4-μm thickness sections according to Pedrycz and Czerny [30]. For negative controls, the primary antibody was omitted. In brief, mouse anti-caspase-3 (diluted 1:250, Santa Cruz Biotechnology, USA), was incubated with sections for 60 minutes. Primary antibodies were diluted in Tris buffered saline (TBS)/1% bovine serum albumin (BSA). Then a biotinylated secondary antibody directed against mice immunoglobulin (Biotinylated Link Universal – DakoCytomation kit, supplied ready to use) was added and incubated for 15 minutes, followed by addition of horse radish peroxidase conjugated with streptavidin (DakoCytomation kit, supplied ready to use) also incubated for 15 minutes. At the sites of immunolocalization of the primary antibodies, a reddish to brown color appeared after adding 3-amino-9-ethylcarbasole (AEC) (DakoCytomationkit, supplied ready to use) for 15 minutes. Specimens were counterstained with hematoxylin for one minute and mounted using the Aquatex fluid (Merck KGaA, Germany).

### Statistical analysis

The obtained data were presented as means ± standard error. Statistical analysis was performed using an unpaired Student’s t-test using a statistical package program (SPSS version 17.0). Differences among groups were considered significant at *p* < 0.05.

### Ethics committee approval

The present study followed the European Community Directive (86/609/EEC) and national rules on animal care that are in accordance with the NIH *Guide for the Care and Use of Laboratory Animals* (available at http://grants.nih.gov/grants/olaw/Guide-for-the-care-and-use-of-laboratory-animals.pdf).

## Results

Changes in levels of serum parameters affected by the single IP injection of crude venom of *N. haje* after four hours are shown in Table 1. Levels of AST, γGT and total billirubin were significantly increased (*p* < 0.05) when compared to untreated rats. However, the level of ALT was non-significantly changed. In addition, the total serum protein level was significantly increased (38.6% at *p* < 0.05) four hours after *N. haje* venom injection.

**Table 1:** **Changes in liver function of rats induced by** ***Naja haje*** **venom after four hours**

Parameters	Control rats	Intoxicated rats
Serum ALT (U/mL)	70.13 ± 2.17	75.26 ± 1.07
Serum AST (U/mL)	56.08 ± 0.74	95.50 ± 0.32*
Serum γGT (U/L)	3.88 ± 0.65	4.32 ± 0.32*
Serum ALP (IU/L)	55.40 ± 5.94	147.73 ± 8.97*
Serum TB (mg/dL)	0.64 ± 0.21	1.94 ± 0 .53*
Serum protein (mg/ dL)	7.20 ± 0.26	9.98 ± 0.40*

*significant changes at *p* < 0.05 with respect to the control group; values are means ± SE (n = 6).

ALT: alanine aminotransferase, AST: aspartate aminotransferase, γGT: γ-glutamyl transpeptidase, ALP: alkaline phosphatase, TB: total bilirubin.

**Figure 1:**
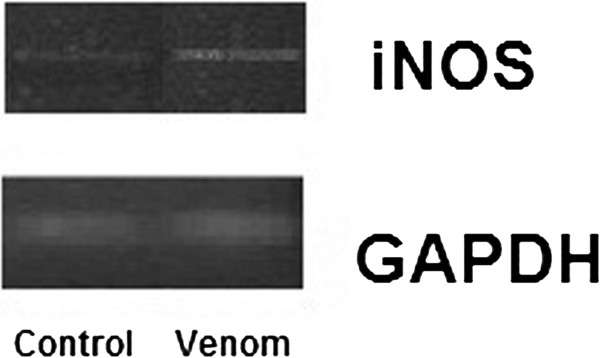
**Change in the expressions of inducible nitric oxide synthase (iNOS) in the liver of male rats injected with** ***Naja haje*** **crude venom.** The expression of the tested enzyme was normalized by comparison with the expression of GAPDH in each sample.

To check the oxidative stress status in liver in response to *N. haje* crude venom, we measured LPO levels in serum and liver homogenate. Results are displayed in Table 2 and showed that crude venom induced increment in LPO production and NO generation in both serum and liver homogenate. In addition, NO generation in serum and liver increased significantly by 42.97% and 49.23%, respectively, when compared to control rats (Table 2). Through RT-PCR analysis (Figure 1) an increase in iNOS expression was evident only in the envenomated group. iNOS transcription, however, was confirmed to accompany the previously observed increase in NO content in the envenomated group. iNOS transcription, however, was confirmed to accompany the previously observed increase in NO content in the envenomated group.

**Table 2:** **Levels of serum and liver lipid peroxidation (LPO) and nitrite/nitrate (NO) of rats induced by** ***Naja haje*** **snake venom after four hours**

Parameters	Control rats	Intoxicated rats
Serum LPO (nmol/mL)	32.37 ± 1.50	43.55 ± 1.05*
Liver LPO (nmol/g tissue)	1027.20 ± 27.07	1220.34 ± 40.09*
Serum NO (μmol/L)	47.33 ± 4.03	67.67 ± 3.60*
Liver NO (μmol/g tissue)	128.54 ± 5.86	191.82 ± 15.49*

*significant change at *p* < 0.05 with respect to the control group; values are means ± SE (n = 6).

In order to investigate the responses of the reactive oxygen scavenging system of liver tissue after four hours of exposure to *N. haje* venom, the level and the activity of enzymatic/non-enzymatic antioxidant system were measured (Table 3). The levels of GSH in serum and liver homogenate were significantly decreased (*p* < 0.05) by 35.26% and 38.66%, respectively. Moreover, activities of enzymatic antioxidant system were significantly diminished (*p* < 0.05) in the liver, where the activity of GR dropped by 40.72%. Additionally, GST diminished by 60.66% and CAT activity was decreased by 68.29% regarding control animals. On the other hand, the SOD and GPx activities were found to be significantly increased (*p* < 0.05) in liver tissue. SOD augmented from 1.06 ± 0.03 in control (untreated rats) to 1.72 ± 0.08 U/g of tissue in treated rats with *N. haje* crude venom (Table 3).

**Table 3:** **Changes in antioxidant state of rats induced by** ***Naja haje*** **snake venom after four hours**

Parameters	Control rats	Intoxicated rats
Serum GSH (mmol/mL)	1.56 ± 0.34	1.01 ± 0.15*
Liver GSH (mmol/g tissue)	92.42 ± 17.07	56.69 ± 2.46*
Liver GPx (U/g tissue)	1722.43 ± 69.54	2253.41 ± 71.24*
Liver GR (μmol/h/g tissue)	93.78 ± 10.75	55.59 ± 18.27*
Liver GST (μmol/h/g tissue)	0.61 ± 0.02	0.24 ± 0.01*
Liver SOD (U/g tissue)	1.06 ± 0.03	1.72 ± 0.08*
Liver CAT (U/g tissue)	0.41 ± 0.02	0.13 ± 0.01*

*significant change at *p* < 0.05 with respect to the control group; values are means ± SE (n = 6).

GSH: glutathione, GPx: glutathione peroxidase, GR: glutathione reductase, SOD: superoxide dismutase, CAT: catalase.

**Figure 2:**
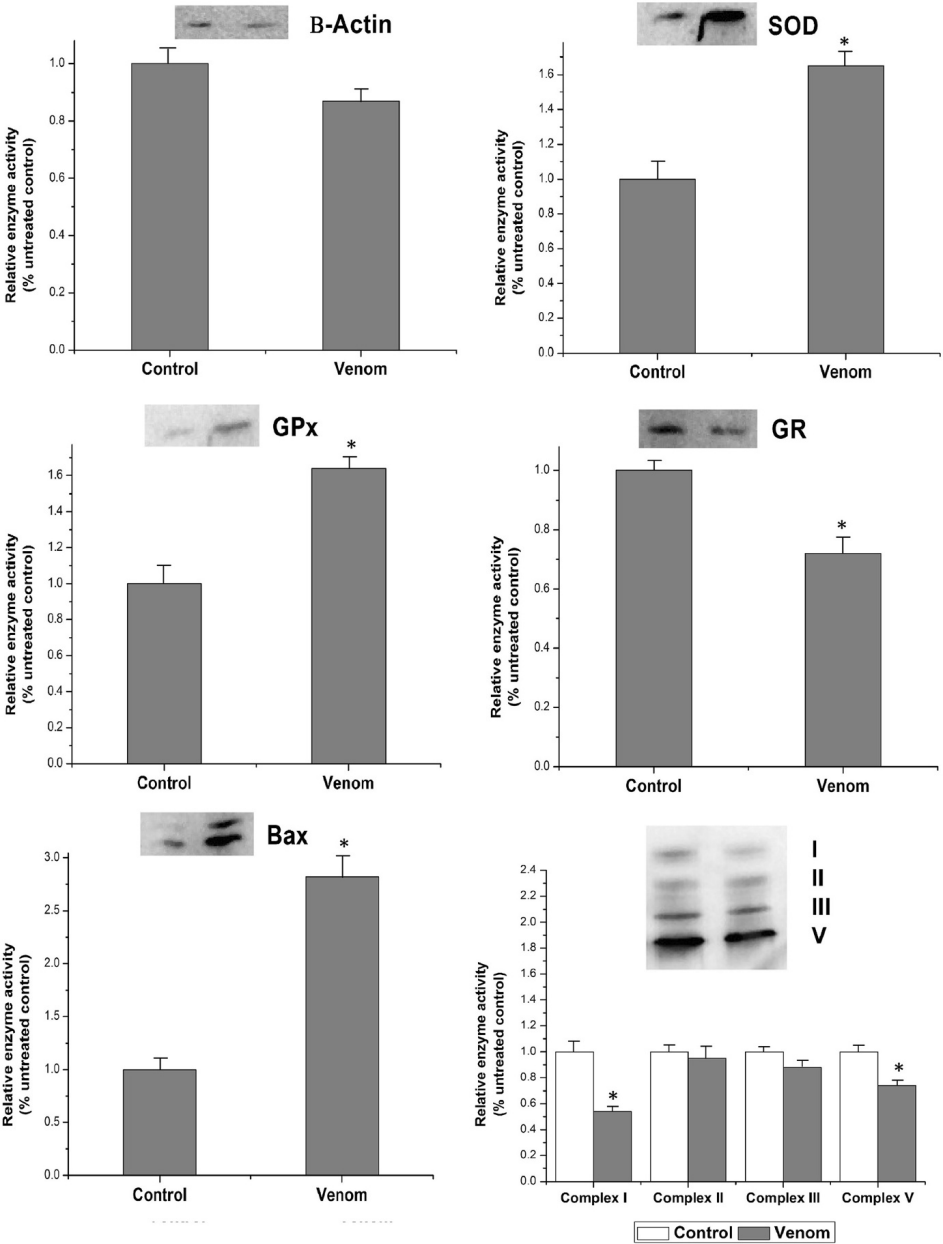
**Expressions of** **β-actin, SOD, GPx, GR Bax, Bcl-2 and mitochondrial respiratory complexes proteins in the liver of rats injected with** ***Naja haje*** **crude venom.** Values are means ± SD (n = 6). *Significant change at *p* < 0.05 regarding the control group.

SOD, GPx, and GR expression in tested tissue was modulated by *N. haje* crude venom. As observed by densitometry, the reduction of GR in envenomated rats was 38% in comparison with controls (Figure 2). SOD and GPx protein levels were also higher in the envenomated group. These results were supported by the activities of the corresponding enzymes (Table 3).

Figure 2 shows alterations in the activity of mitochondrial respiratory complexes. Complex II, III, III and V activities in the examined livers of envenomated rats were decreased by 56%, 5%, 12% and 26%, respectively, *p* < 0.05 when compared to the controls.

Effects of *N. haje* crude venom on Bax and Bcl-2 protein content in the liver are presented in Figure 2. *N. haje* venom injection caused a significant (*p* < 0.05) increase in Bax and a significant decrease in Bcl-2 protein content (*p* < 0.05).

**Figure 3:**
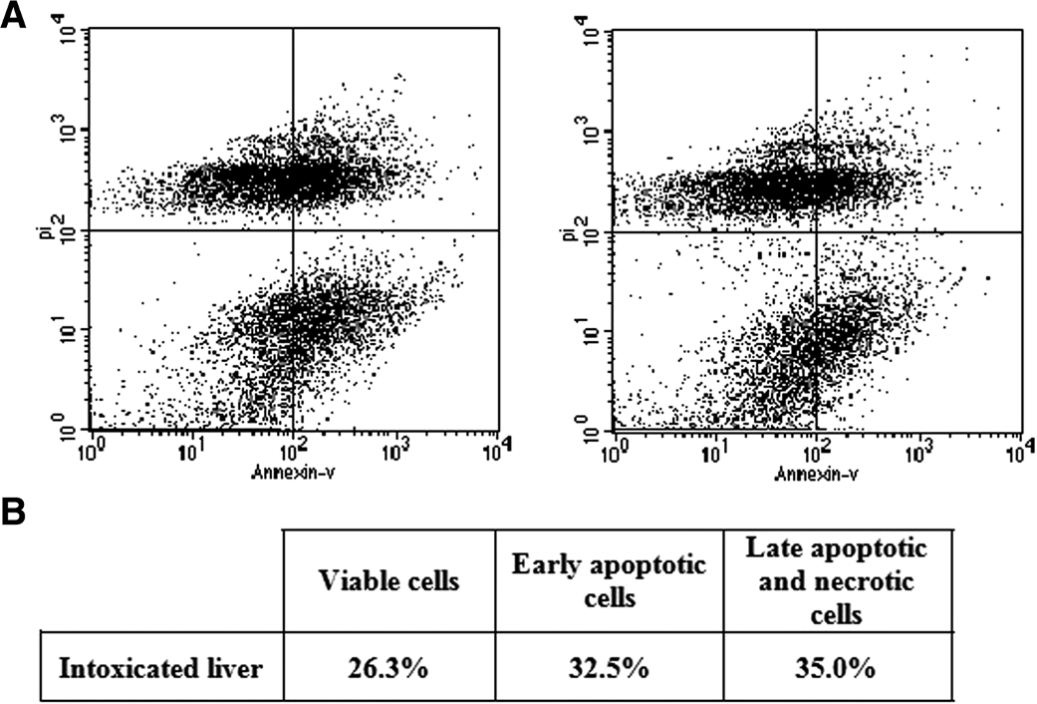
**Assessment of apoptosis in hepatic tissue of male rats injected with** ***Naja haje*** **crude venom. (A)** Representative flow cytometry dot plot of FITC-annexin V/propidium iodide. **(B)** Table showing: viable cells, early apoptotic cells, and late apoptotic and necrotic cells.

Hepatocytes were stained with both propodium iodide (PI) and fluorescein isothiocyanate (FITC)-labeled annexin V (AV-FITC) in order to enable the analysis of apoptotic cells with flow cytometry. Necrotic cells were demonstrated by AV–/PI + or AV+/PI + staining, because when membrane integrity is lost PI enters cells and combines with nucleic acids. Early apoptotic cells were demonstrated by AV+/PI– staining, because when AV combines with phosphatidylserine, they translocate to the outer leaflet of the plasma membrane during apoptosis. AV+/PI + stained cells were likely to be late apoptotic or necrotic cells whereas AV–/PI– cells represented viable cells. In the control group, most liver cells were viable (Figure 3). When rats were exposed to *N. haje* venom, the number of AV + cells were significantly increased (67.5%, *p* < 0.05).

**Figure 4:**
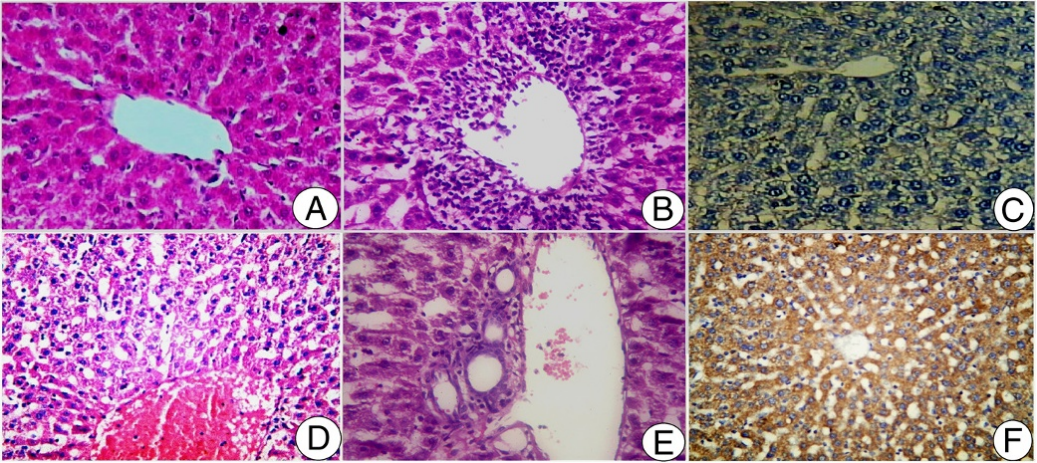
**Histological and immunohistochemical investigations in the livers of rats. (A)** Control liver section. **(B, D, E)** *Naja haje* group liver sections (HE stain, 400×). **(C)** Section from control group showing low affinity to caspase-3. **(F)** Section from the group treated with *Naja haje* crude venom showing high affinity to caspase-3.

Liver section of control animals (Figure 4A) revealed with normal cell structure, while liver sections of rats injected with LD_50_ of *N. haje* crude venom showed inflammatory cell infiltration around the hepatic vein, distended blood sinusoids, hepatocyte vacuolation and prominent van Kupffer cells (Figure 4B). Severe necrosis and apoptosis were also seen (Figure 4D). Figure 4E shows severe congestion in the central vein. Immunohistochemical investigations for caspase-3 in hepatocytes of the control and envenomated groups are represented in Figure 4C and F. In the envenomated group, the number of caspase-3 positive immunostaining hepatocytes was significantly increased, which proves the pro-apoptotic activity of *N. haje* crude venom (Figure 4F).

## Discussion

Several studies analyzing snake venom effects on animal cells – from blood, marrow, muscle, liver, kidney and skin – showed different results, depending on the experimental concentrations, exposure time, site of injection, and the type of toxin [31, 32]. The liver is a major producer of most serum proteins and their total levels in the blood are regulated by the liver function. In the present study, the elevation of ALT, AST, γGT, ALP, and total bilirubin in envenomated rats could be attributed to the hepatocyte damage. Such imbalance has been reported by other researchers who analyzed snake venom effects [33–35].

The mechanism by which *N. haje* venom induces cytotoxic effects is still not clear. To the best of our knowledge, there are no available data regarding the involvement of oxidative stress induced by *N. haje* venom exposure *in vitro*. In order to evaluate the ability of *N. haje* venom to produce oxidative stress, we choose to monitor one of the earliest responses of oxidative stress, which is the increase of stress markers in liver homogenates.

Levels of early markers of oxidative stress, including antioxidant enzymes, may be altered in the presence of lower levels of oxidative stress. To this end, we have monitored antioxidant enzyme activities. Our results clearly showed that *N. haje* venom enhances SOD and GPx activities (Table 3). The induction of the enzymatic antioxidant defenses after the exposure to *N. haje* venom could be considered as an adaptive response; that is, a compensatory mechanism that enables cells to overcome the damage caused.

To further demonstrate the implication of oxidative stress in venom induced toxicity, we decided to monitor LPO. Lipid peroxidation is one of the suggested cytotoxic mechanisms of different venoms. The MDA is an end product of lipid peroxidation, considered as a late biomarker of oxidative stress and cellular damage [36]. It is generally considered as an excellent indicator of lipid peroxidation [37]. We have shown an increase of lipid peroxidation level that seemed related to *N. haje* crude venom as inferred by the amount of MDA generated, confirming an increase of free radicals production. This fact emphasizes that the oxidative damage is induced by the venom in the liver of rats (Table 2).

In addition, venom phospholipase caused a disturbance of the cell membrane permeability with consequent influx of Na^+^ and water [38]. Chethankumar and Srinivas [39] concluded that the exposure of cellular membranes to *N. haje* venom phospholipase significantly decreased the Na^+^/K^+^ ATPase activities, thereby altering the ionic gradients, disorganizing the membrane lipid bilayer and eventually leading to cell death. According to Mukherjee and Maity [40], the progression of hepatic cellular swelling together with the effect of the venom phospholipase on the membranous phospholipids during envenomation might be among the factors responsible for the rupture of hepatic cell membranes and the occurrence of the observed cellular damage in the present study.

L-amino acid oxidases (LAAOs) are flavoproteins that are able to catalyze the oxidative deamination of L-amino acids to produce the corresponding α-keto acids along with the concomitant release of hydrogen peroxide (H_2_O_2_) and ammonia. Although they occur in many different organisms from invertebrates to vertebrates, their functions *in vivo* are uncertain. LAAO is widely distributed in venomous snakes including the viperids and elapids and is thought to contribute to their toxicity, possibly through H_2_O_2_ formed as a result of reoxidation of the transiently reduced FAD cofactor by molecular oxygen [41, 42]. The enzyme is the major component of snake venoms, and in some species this enzyme alone constitutes approximately 30% of the total protein content [42, 43]. Furthermore, venom LAAO has been shown to induce cell death in several mammalian cell lines [44]. The effect was attributed to the formation of localized high concentrations of H_2_O_2_, a known reactive oxygen species (ROS). It is interesting to note that the LAAO-induced apoptosis has been reported to be different from that caused by exogenous H_2_O_2_, suggesting that the mode of delivery of H_2_O_2_ is an important factor. In addition, snake venom LAAOs appear to be cytotoxic against many organisms [45].

Tempone *et al.* [46] suggested that cells submitted to oxidative stress induced by LAAO generated H_2_O_2_ that could activate heat shock proteins and initiate cell membrane disorganization, DNA fragmentation, apoptosis and therefore cell death. Sun *et al.* [47] suggested that the generated peroxide activates the transcription of such factors as the nuclear factor B, the activator protein 1, Fas/Apo-1 and p53.

Apoptosis is an extremely complex and sophisticated process, involving many events, including the expression of apoptosis-related genes. In general, apoptosis is a three-stage process that includes initiation, effector and degradation periods. The initiation phase is largely dependent on cell type and apoptotic stimulus (e.g., oxidative stress, DNA damage, etc.). During the initiation phase, specific pro-apoptotic signal transduction pathways or non-specific damage pathways are activated. In certain instances, initiation phase may influence the efficacy of the effector and/or degradation phases. In the effector phase, there is activation of proteases, nucleases, and other diffusible intermediaries that participate in the degradation phase of DNA. Together, the effector and degradation phases promote the ultrastructural features that are suggestive of apoptosis. Finally, these steps are followed by rapid engulfment of the deceased cell by neighboring phagocytic cells [48].

Internal and external mitochondrial membrane permeability (MMP) changes led to disappearance of MMP and release of cytochrome c and other pro-apoptotic factors into the cytosol. The release of pro-apoptotic factors in the cytoplasm may initiate apoptosis cascade reaction, which includes activation of caspase-3 and other substances that trigger proteolytic enzymes and break DNA into fragments [49]. Our data revealed that *N. haje* snake venom induces apoptosis in hepatocytes through increased transcription of caspase-3 gene. These results suggest that *N. haje* venom components may increase expression of certain pro-apoptotic genes that lead to cell apoptosis.

## Conclusion

In conclusion, despite advances in our understanding of the hepatotoxicity response to *N. haje* venom, much remains to be learned on the mechanisms involved in the initiation and development of the hepatotoxicity events triggered by this venom. Particularly regarding the range of mediators involved, the regulatory steps associated with their production and action, and the actual types and subtypes of receptors activated by the main mediators. A deficit in our study is the usage of crude venom, therefore it is difficult to establish which component lead to our results.

## Abbreviations

CAT: Catalase
GPx: Glutathione peroxidase
GR: Glutathione reductase
GSH: Glutathione
GST: Glutathione-S-transferase
LPO: Lipid peroxidation
MDA: Malondialdehyde
NO: Nitric oxide
ROS: Reactive oxygen species
SOD: Superoxide dismutase.
